# Supplementary figures and images for: Impact of maternal protein restriction on Hypoxia-Inducible Factor (HIF) expression in male fetal kidney development
Source: PLoS One. 2023 May 4;18(5):e0266293. doi: 10.1371/journal.pone.0266293 (PMC10159110; doi:10.1371/journal.pone.0266293)

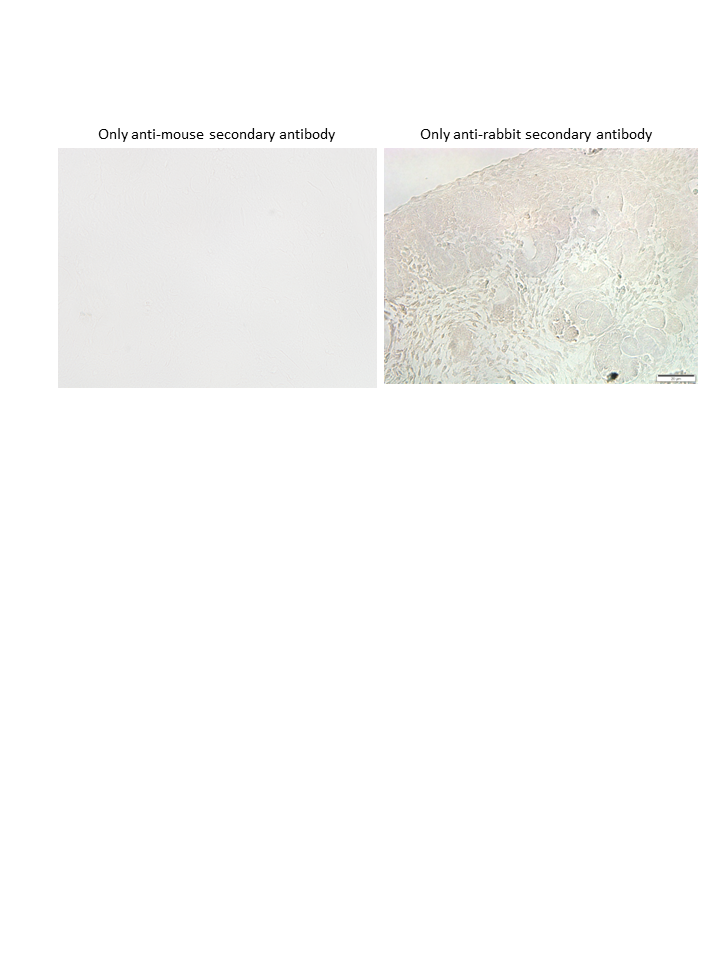

Supplement: S1 Fig — (TIF) [file pone.0266293.s002.TIF]
