# Supplementary material for: Impact of maternal protein restriction on Hypoxia-Inducible Factor (HIF) expression in male fetal kidney development
Source: PLoS One. 2023 May 4;18(5):e0266293. doi: 10.1371/journal.pone.0266293 (PMC10159110; doi:10.1371/journal.pone.0266293)
Supplement: S1 File — (PDF) [file pone.0266293.s003.pdf]

Full Arrive 2.0 Files

1. *Study Design*

The experiments were conducted as described in detail previously [Mesquita et al., 2010a,b] on age-matched female and male rats of sibling-mated Wistar *HanUnib* rats (250–300 g) originated from a breeding stock supplied by CEMIB/ UNICAMP, Campinas, SP, Brazil. The environment and housing presented the right conditions for managing their health and well-being during the experimental procedure. Immediately after weaning at three weeks of age, animals were maintained under controlled temperature (25°C) and lighting conditions (07:00–19:00h) with free access to tap water and standard laboratory rodent chow (Purina Nuvital, Curitiba, PR, Brazil: Na+ content: 135 ± 3µEq/g; K+ content: 293 ± 5µEq/g), for 12 weeks before breeding. The Institutional Ethics Committee on Animal Use at São Paulo State University (#446-CEUA/UNESP) approved the experimental protocol. The general guidelines established by the Brazilian College of Animal Experimentation were followed throughout the investigation. Four females and one male were kept in the same cage for two hours in the dark cycle, and this day 1 of pregnancy was designated the day on which the vaginal smear exhibited sperm. Then, dams were maintained ad libitum throughout the entire pregnancy on an isocaloric rodent laboratory chow with either standard protein content [NP, n = 10] (17% protein) or low protein content [LP, n = 10] (6% protein). At 17 days of gestation (17-DG), the dams were anesthetized by ketamine (75mg/kg) and xylazine (10mg/kg), and the uterus was exposed. The fetuses were randomly collected, including their position in the uterine horns, at the close time, at 17 gestational days for both experimental groups. An equal number of the fetus was taken from both uteri horn sides. The fetuses were removed and immediately euthanized by decapitation. The fetuses were weighed, and the tail and limbs were collected for sexing. Male fetuses from half of the dams (5 for each group) metanephros was isolated and collected for RT-qPCR, and the other half of fetuses were immersion fixed for immunohistochemistry analyses. Each litter was considered n = 1 to prevent litter effects from biasing or data analysis, and only one pup per litter was used for each experiment.

Sexing determination - The present study was performed only in male 17-GD progeny, and the sexing was determined by Sry conventional PCR (Polymerase Chain Reaction) sequence analysis. The DNA was extracted by enzymatic lysis with proteinase K and Phenol-

Chloroform. The Master Mix Colorless—Promega was used for reaction with the manufacturer's cycling conditions. The Integrated DNA Technologies (IDT) synthesized the primer following sequences below:

1. Forward: 5'-TACAGCCTGAGGACATATTA-3'
2. Reverse: 5'-GCACTTTAACCCTTCGATTAG-3'.

It is essential to state here that sex hormones determine sexual phenotype dimorphism in the fetal-programmed disease model in adulthood by changes in the long-term control of neural, cardiac, and endocrine functions. Thus, the present study was limited and performed on male rats considering the findings above to eliminate interferences due to gender differences [5-7].

References

Mesquita FF, Gontijo JA, Boer PA. Expression of renin-angiotensin system signalling compounds in maternal protein-restricted rats: effect on renal sodium excretion and blood pressure. Nephrol Dial Transplant 2010a; 25, 380-388. DOI: 10.1093/ndt/gfp505.

Mesquita FF, Gontijo JAR, Boer PA. Maternal undernutrition and the offspring kidney: from fetal to adult life. *Braz J Med Biol Res 2010b;* 2010b; 43, 1010–1018. DOI: 10.1590/s0100-879x2010007500113

Sene LB, Mesquita FF, de Moraes LN, Santos DC, Carvalho R, Gontijo JA, Boer PA. Involvement of renal corpuscle microRNA expression on epithelial-to-mesenchymal transition in maternal low protein diet in adult programmed rats. PLoS One. 2013; 19: e71310. https://doi.org/10.1371/journal.pone. 0071310 PMID: 23977013

Sene LB, Rizzi VHG, Gontijo JAR, Boer PA. (2018). Gestational low-protein intake enhances whole-kidney miR-192 and miR-200 family expression and epithelial-to-mesenchymal transition in rat adult male offspring. *J Exp Biol* 2018; 221:jeb171694. DOI: 10.1242/jeb.171694

Sene LB, Scarano WR, Zapparoli A, Gontijo JAR, Boer PA. Impact of gestational low-protein intake on embryonic kidney microRNA expression and in nephron progenitor cells of the male fetus. *PLoS One* 2021; 16:e0246289. DOI: 10.1371/journal.pone.0246289

Lamana GL, Ferrari ALL, Gontijo JAR, Boer PA. Gestational and breastfeeding low-protein intake on blood pressure, kidney structure, and renal function in male rat offspring in adulthood. *Front. Physiol 2021;* 12:658431 DOI: 10.3389/fphys.2021.658431

2. <u>*Sample Size*</u>

Total RNA Extraction - Isolated two-kidney tissue RNA pool was extracted from one fetus of each litter of the NP (n = 5) and LP (n = 5) offspring using Trizol reagent (Invitrogen), according to the instructions specified by the manufacturer.

After centrifugation, the material is separated into 3 phases, (a) the upper phase is aqueous and transparent; (b) the intermediate phase, and (c) the reddish lower organic phase. The RNA remains in the aqueous phase and is recovered through precipitation, carried out by washing isopropyl alcohol cycles and centrifugation. Total RNA quantity was determined by the absorbance at 260 nm using a nanoVue spectrophotometer (GE Healthcare, USA), and the RNA purity was assessed by the A 260 nm/A 280 nm and A 260 nm/A 230 nm ratios (acceptable when both ratios were >1.8). RNA Integrity was ensured by obtaining an RNA Integrity Number - RIN >8 with Agilent 2100 Bioanalyzer (Agilent Technologies, Germany).

Immunohistochemistry – The fetus (n = 5 per group) was removed and immediately fixed in 4% paraformaldehyde (0.1 M phosphate, pH 7.4). The materials were dehydrated, diaphanized, and included in paraplast, and the blocks were cut into 5-µm-thickness sections. For immunohistochemistry, the paraffin sections were hydrated and washed in PBS (pH 7.2), and then the antigenic recovery was made with citrate buffer pH 6.0 for 25 minutes in the pressure cooker. Endogenous peroxidase was blocked with hydrogen peroxide and methanol for 10 minutes. For non-specific binding, the slides were incubated with a blocking solution (5% skimmed milk powder, in PBS) for 1 hour. The sections were incubated with the primary antibody (Table 2) and diluted in 1% BSA overnight in the refrigerator. After washing with PBS, the sections were exposed to the specific secondary antibody for 2 hours at room temperature. The slides were washed with PBS. The slices were revealed with DAB (3,3’-diaminobenzidine tetrahydrochloride, Sigma—Aldrich CO®, USA). After successive washing in running water, the slides were counterstained with hematoxylin, dehydrated, and mounted with a coverslip using Entellan®. When the proteins studied had nuclear localization, the slides were not counterstained with hematoxylin in order not to cover the labeling. No immunoreactivity was seen in control experiments in which one of the primary antibodies was omitted Figure 1S.

References

Mesquita FF, Gontijo JA, Boer PA. Expression of renin-angiotensin system signalling compounds in maternal protein-restricted rats: effect on renal sodium excretion and blood pressure. Nephrol Dial Transplant 2010a; 25, 380-388. DOI: 10.1093/ndt/gfp505.

Mesquita FF, Gontijo JAR, Boer PA. Maternal undernutrition and the offspring kidney: from fetal to adult life. *Braz J Med Biol Res 2010b;* 2010b; 43, 1010–1018. DOI: 10.1590/s0100-879x2010007500113

Sene LB, Mesquita FF, de Moraes LN, Santos DC, Carvalho R, Gontijo JA, Boer PA. Involvement of renal corpuscle microRNA expression on epithelial-to-mesenchymal transition in maternal low protein diet in adult programmed rats. PLoS One. 2013; 19: e71310. https://doi.org/10.1371/journal.pone. 0071310 PMID: 23977013

Sene LB, Rizzi VHG, Gontijo JAR, Boer PA. (2018). Gestational low-protein intake enhances whole-kidney miR-192 and miR-200 family expression and epithelial-to-mesenchymal transition in rat adult male offspring. *J Exp Biol* 2018; 221:jeb171694. DOI: 10.1242/jeb.171694

Sene LB, Scarano WR, Zapparoli A, Gontijo JAR, Boer PA. Impact of gestational low-protein intake on embryonic kidney microRNA expression and in nephron progenitor cells of the male fetus. *PLoS One* 2021; 16:e0246289. DOI: 10.1371/journal.pone.0246289

Lamana GL, Ferrari ALL, Gontijo JAR, Boer PA. Gestational and breastfeeding low-protein intake on blood pressure, kidney structure, and renal function in male rat offspring in adulthood. *Front. Physiol 2021;* 12:658431 DOI: 10.3389/fphys.2021.658431

3. <u>Inclusion and exclusion criteria</u>

The fetuses were randomly collected, including their position in the uterine horns, at the close time, at 17 gestational days for both experimental groups. An equal number of the fetus was taken from both uteri horn sides. The fetuses were removed and immediately euthanized by decapitation. The fetuses were weighed, and the tail and limbs were collected for sexing. Male fetuses from half of the dams (5 for each group) metanephros was isolated and collected for RT-qPCR, and the other half of fetuses were immersion fixed for immunohistochemistry analyses. Each litter was considered n = 1 to prevent litter effects from biasing or data analysis, and only one pup per litter was used for each experiment.

The Welch test was performed in situations of heteroscedasticity, when a large variance was observed between groups studied.

References

Mesquita FF, Gontijo JA, Boer PA. Expression of renin-angiotensin system signalling compounds in maternal protein-restricted rats: effect on renal sodium excretion and blood pressure. Nephrol Dial Transplant 2010a; 25, 380-388. DOI: 10.1093/ndt/gfp505.

Mesquita FF, Gontijo JAR, Boer PA. Maternal undernutrition and the offspring kidney: from fetal to adult life. *Braz J Med Biol Res 2010b;* 2010b; 43, 1010–1018. DOI: 10.1590/s0100-879x2010007500113

Sene LB, Mesquita FF, de Moraes LN, Santos DC, Carvalho R, Gontijo JA, Boer PA. Involvement of renal corpuscle microRNA expression on epithelial-to-mesenchymal transition in maternal low protein diet in adult programmed rats. PLoS One. 2013; 19: e71310. https://doi.org/10.1371/journal.pone. 0071310 PMID: 23977013

Sene LB, Rizzi VHG, Gontijo JAR, Boer PA. (2018). Gestational low-protein intake enhances whole-kidney miR-192 and miR-200 family expression and epithelial-to-mesenchymal transition in rat adult male offspring. *J Exp Biol* 2018; 221:jeb171694. DOI: 10.1242/jeb.171694

Sene LB, Scarano WR, Zapparoli A, Gontijo JAR, Boer PA. Impact of gestational low-protein intake on embryonic kidney microRNA expression and in nephron progenitor cells of the male fetus. *PLoS One* 2021; 16:e0246289. DOI: 10.1371/journal.pone.0246289

Lamana GL, Ferrari ALL, Gontijo JAR, Boer PA. Gestational and breastfeeding low-protein intake on blood pressure, kidney structure, and renal function in male rat offspring in adulthood. *Front. Physiol 2021;* 12:658431 DOI: 10.3389/fphys.2021.658431

4. *Randomization*

The fetuses were randomly collected, including their position in the uterine horns, at the close time, at 17 gestational days for both experimental groups. An equal number of the fetus was taken from both uteri horn sides.

References

Mesquita FF, Gontijo JA, Boer PA. Expression of renin-angiotensin system signalling compounds in maternal protein-restricted rats: effect on renal sodium excretion and blood pressure. Nephrol Dial Transplant 2010a; 25, 380-388. DOI: 10.1093/ndt/gfp505.

Mesquita FF, Gontijo JAR, Boer PA. Maternal undernutrition and the offspring kidney: from fetal to adult life. *Braz J Med Biol Res 2010b;* 2010b; 43, 1010–1018. DOI: 10.1590/s0100-879x2010007500113

Sene LB, Mesquita FF, de Moraes LN, Santos DC, Carvalho R, Gontijo JA, Boer PA. Involvement of renal corpuscle microRNA expression on epithelial-to-mesenchymal transition in maternal low protein diet in adult programmed rats. PLoS One. 2013; 19: e71310. https://doi.org/10.1371/journal.pone. 0071310 PMID: 23977013

Sene LB, Rizzi VHG, Gontijo JAR, Boer PA. (2018). Gestational low-protein intake enhances whole-kidney miR-192 and miR-200 family expression and epithelial-to-mesenchymal transition in rat adult male offspring. *J Exp Biol* 2018; 221:jeb171694. DOI: 10.1242/jeb.171694

Sene LB, Scarano WR, Zapparoli A, Gontijo JAR, Boer PA. Impact of gestational low-protein intake on embryonic kidney microRNA expression and in nephron progenitor cells of the male fetus. *PLoS One* 2021; 16:e0246289. DOI: 10.1371/journal.pone.0246289

Lamana GL, Ferrari ALL, Gontijo JAR, Boer PA. Gestational and breastfeeding low-protein intake on blood pressure, kidney structure, and renal function in male rat offspring in adulthood. *Front. Physiol 2021;* 12:658431 DOI: 10.3389/fphys.2021.658431

5. *Bliding*

The immunohistochemistry quantification was blinded performed, by microscopic fields digitized (Olympus BX51) using CellSens Dimension. The studies were performed in a blinded and similar way for both groups of animals (NP and LP). In the metanephros section at its greatest longitudinal extent (% established for each group), all the CAP area was delimited, and the percentage of the marked area was automatically calculated.

References

Mesquita FF, Gontijo JA, Boer PA. Expression of renin-angiotensin system signalling compounds in maternal protein-restricted rats: effect on renal sodium excretion and blood pressure. Nephrol Dial Transplant 2010a; 25, 380-388. DOI: 10.1093/ndt/gfp505.

Mesquita FF, Gontijo JAR, Boer PA. Maternal undernutrition and the offspring kidney: from fetal to adult life. *Braz J Med Biol Res 2010b;* 2010b; 43, 1010–1018. DOI: 10.1590/s0100-879x2010007500113

Sene LB, Mesquita FF, de Moraes LN, Santos DC, Carvalho R, Gontijo JA, Boer PA. Involvement of renal corpuscle microRNA expression on epithelial-to-mesenchymal transition in maternal low protein diet in adult programmed rats. PLoS One. 2013; 19: e71310. https://doi.org/10.1371/journal.pone. 0071310 PMID: 23977013

Sene LB, Rizzi VHG, Gontijo JAR, Boer PA. (2018). Gestational low-protein intake enhances whole-kidney miR-192 and miR-200 family expression and epithelial-to-mesenchymal transition in rat adult male offspring. *J Exp Biol* 2018; 221:jeb171694. DOI: 10.1242/jeb.171694

Sene LB, Scarano WR, Zapparoli A, Gontijo JAR, Boer PA. Impact of gestational low-protein intake on embryonic kidney microRNA expression and in nephron progenitor cells of the male fetus. *PLoS One* 2021; 16:e0246289. DOI: 10.1371/journal.pone.0246289

Lamana GL, Ferrari ALL, Gontijo JAR, Boer PA. Gestational and breastfeeding low-protein intake on blood pressure, kidney structure, and renal function in male rat offspring in adulthood. *Front. Physiol 2021;* 12:658431 DOI: 10.3389/fphys.2021.658431

6. *Outcome measures*

The fetuses were randomly collected, including their position in the uterine horns, at the close time, at 17 gestational days for both experimental groups. An equal number of the fetus was taken from both uteri horn sides. The fetuses were removed and immediately euthanized by decapitation. The fetuses were weighed, and the tail and limbs were collected for sexing. Male fetuses from half of the dams (5 for each group) metanephros was isolated and collected for RT-qPCR, and the other half of fetuses were immersion fixed for immunohistochemistry analyses. Each litter was considered n = 1 to prevent litter effects from biasing or data analysis, and only one pup per litter was used for each experiment.

References

Mesquita FF, Gontijo JA, Boer PA. Expression of renin-angiotensin system signalling compounds in maternal protein-restricted rats: effect on renal sodium excretion and blood pressure. Nephrol Dial Transplant 2010a; 25, 380-388. DOI: 10.1093/ndt/gfp505.

Mesquita FF, Gontijo JAR, Boer PA. Maternal undernutrition and the offspring kidney: from fetal to adult life. *Braz J Med Biol Res 2010b;* 2010b; 43, 1010–1018. DOI: 10.1590/s0100-879x2010007500113

Sene LB, Mesquita FF, de Moraes LN, Santos DC, Carvalho R, Gontijo JA, Boer PA. Involvement of renal corpuscle microRNA expression on epithelial-to-mesenchymal transition in maternal low protein diet in adult programmed rats. PLoS One. 2013; 19: e71310. https://doi.org/10.1371/journal.pone. 0071310 PMID: 23977013

Sene LB, Rizzi VHG, Gontijo JAR, Boer PA. (2018). Gestational low-protein intake enhances whole-kidney miR-192 and miR-200 family expression and epithelial-to-mesenchymal transition in rat adult male offspring. *J Exp Biol* 2018; 221:jeb171694. DOI: 10.1242/jeb.171694

Sene LB, Scarano WR, Zapparoli A, Gontijo JAR, Boer PA. Impact of gestational low-protein intake on embryonic kidney microRNA expression and in nephron progenitor cells of the male fetus. *PLoS One* 2021; 16:e0246289. DOI: 10.1371/journal.pone.0246289

Lamana GL, Ferrari ALL, Gontijo JAR, Boer PA. Gestational and breastfeeding low-protein intake on blood pressure, kidney structure, and renal function in male rat offspring in adulthood. *Front. Physiol 2021;* 12:658431 DOI: 10.3389/fphys.2021.658431

7. *Statistical methods*

Statistical Analysis - Data was previously tested to assess the normality of distribution frequency and equality of variance by the Shapiro-Wilk and the Levene test. Data are expressed as the mean ± standard deviation (SD). Comparisons between two groups were performed using Student's t-test when data were normally distributed and the Mann-Whitney test when distributions were non-normal. Comparisons between two groups through the weeks were performed using 2-way ANOVA for repeated measurements test, in which the first factor was the protein content in the pregnant dam's diet and the second factor was time. The mean values were compared using Tukey´s post hoc analysis when the interaction was significant. However, the Welch test was performed in situations of heteroscedasticity, when a large variance was observed between groups studied. Significant differences in the transcriptome were detected using a moderated t-test. GraphPad Prisma

v. 01 software (GraphPad Software, Inc., USA) was used for statistical analysis and graph construction. The significance level was 5%.

References

Mesquita FF, Gontijo JA, Boer PA. Expression of renin-angiotensin system signalling compounds in maternal protein-restricted rats: effect on renal sodium excretion and blood pressure. Nephrol Dial Transplant 2010a; 25, 380-388. DOI: 10.1093/ndt/gfp505.

Mesquita FF, Gontijo JAR, Boer PA. Maternal undernutrition and the offspring kidney: from fetal to adult life. *Braz J Med Biol Res 2010b;* 2010b; 43, 1010–1018. DOI: 10.1590/s0100-879x2010007500113

Sene LB, Mesquita FF, de Moraes LN, Santos DC, Carvalho R, Gontijo JA, Boer PA. Involvement of renal corpuscle microRNA expression on epithelial-to-mesenchymal transition in maternal low protein diet in adult programmed rats. PLoS One. 2013; 19: e71310. https://doi.org/10.1371/journal.pone. 0071310 PMID: 23977013

Sene LB, Rizzi VHG, Gontijo JAR, Boer PA. (2018). Gestational low-protein intake enhances whole-kidney miR-192 and miR-200 family expression and epithelial-to-mesenchymal transition in rat adult male offspring. *J Exp Biol* 2018; 221:jeb171694. DOI: 10.1242/jeb.171694

Sene LB, Scarano WR, Zapparoli A, Gontijo JAR, Boer PA. Impact of gestational low-protein intake on embryonic kidney microRNA expression and in nephron progenitor cells of the male fetus. *PLoS One* 2021; 16:e0246289. DOI: 10.1371/journal.pone.0246289

Lamana GL, Ferrari ALL, Gontijo JAR, Boer PA. Gestational and breastfeeding low-protein intake on blood pressure, kidney structure, and renal function in male rat offspring in adulthood. *Front. Physiol 2021;* 12:658431 DOI: 10.3389/fphys.2021.658431

8. *Experimentation animals*

Animal and Diets **-** The experiments were conducted as described in detail previously [Mesquita et al., 2010a,b] on age-matched female and male rats of sibling-mated Wistar *HanUnib* rats (250–300 g) originated from a breeding stock supplied by CEMIB/ UNICAMP, Campinas, SP, Brazil. The environment and housing presented the right conditions for managing their health and well-being during the experimental procedure.

Immediately after weaning at three weeks of age, animals were maintained under controlled temperature (25°C) and lighting conditions (07:00–19:00h) with free access to tap water and standard laboratory rodent chow (Purina Nuvital, Curitiba, PR, Brazil: Na+ content: 135 ± 3μEq/g; K+ content: 293 ± 5μEq/g), for 12 weeks before breeding.

References

Mesquita FF, Gontijo JA, Boer PA. Expression of renin-angiotensin system signalling compounds in maternal protein-restricted rats: effect on renal sodium excretion and blood pressure. Nephrol Dial Transplant 2010a; 25, 380-388. DOI: 10.1093/ndt/gfp505.

Mesquita FF, Gontijo JAR, Boer PA. Maternal undernutrition and the offspring kidney: from fetal to adult life. *Braz J Med Biol Res 2010b;* 2010b; 43, 1010–1018. DOI: 10.1590/s0100-879x2010007500113

Sene LB, Mesquita FF, de Moraes LN, Santos DC, Carvalho R, Gontijo JA, Boer PA. Involvement of renal corpuscle microRNA expression on epithelial-to-mesenchymal transition in maternal low protein diet in adult programmed rats. PLoS One. 2013; 19: e71310. https://doi.org/10.1371/journal.pone. 0071310 PMID: 23977013

Sene LB, Rizzi VHG, Gontijo JAR, Boer PA. (2018). Gestational low-protein intake enhances whole-kidney miR-192 and miR-200 family expression and epithelial-to-mesenchymal transition in rat adult male offspring. *J Exp Biol* 2018; 221:jeb171694. DOI: 10.1242/jeb.171694

Sene LB, Scarano WR, Zapparoli A, Gontijo JAR, Boer PA. Impact of gestational low-protein intake on embryonic kidney microRNA expression and in nephron progenitor cells of the male fetus. *PLoS One* 2021; 16:e0246289. DOI: 10.1371/journal.pone.0246289

Lamana GL, Ferrari ALL, Gontijo JAR, Boer PA. Gestational and breastfeeding low-protein intake on blood pressure, kidney structure, and renal function in male rat offspring in adulthood. *Front. Physiol 2021;* 12:658431 DOI: 10.3389/fphys.2021.658431

9. Experimental Procedures

The experiments were conducted as described in detail previously [Mesquita et al., 2010a,b] on age-matched female and male rats of sibling-mated Wistar *HanUnib* rats (250–300 g) originated from a breeding stock supplied by CEMIB/ UNICAMP, Campinas, SP, Brazil. The environment and housing presented the right conditions for managing their health and well-

being during the experimental procedure. Immediately after weaning at three weeks of age, animals were maintained under controlled temperature (25°C) and lighting conditions (07:00–19:00h) with free access to tap water and standard laboratory rodent chow (Purina Nuvital, Curitiba, PR, Brazil: Na+ content: 135 ± 3µEq/g; K+ content: 293 ± 5µEq/g), for 12 weeks before breeding. The Institutional Ethics Committee on Animal Use at São Paulo State University (#446-CEUA/UNESP) approved the experimental protocol. The general guidelines established by the Brazilian College of Animal Experimentation were followed throughout the investigation. Four females and one male were kept in the same cage for two hours in the dark cycle, and this day 1 of pregnancy was designated the day on which the vaginal smear exhibited sperm. Then, dams were maintained ad libitum throughout the entire pregnancy on an isocaloric rodent laboratory chow with either standard protein content [NP, n = 10] (17% protein) or low protein content [LP, n = 10] (6% protein). At 17 days of gestation (17-DG), the dams were anesthetized by ketamine (75mg/kg) and xylazine (10mg/kg), and the uterus was exposed. The fetuses were randomly collected, including their position in the uterine horns, at the close time, at 17 gestational days for both experimental groups. An equal number of the fetus was taken from both uteri horn sides. The fetuses were removed and immediately euthanized by decapitation. The fetuses were weighed, and the tail and limbs were collected for sexing. Male fetuses from half of the dams (5 for each group) metanephros was isolated and collected for RT-qPCR, and the other half of fetuses were immersion fixed for immunohistochemistry analyses. Each litter was considered n = 1 to prevent litter effects from biasing or data analysis, and only one pup per litter was used for each experiment.

Sexing determination - The present study was performed only in male 17-GD progeny, and the sexing was determined by Sry conventional PCR (Polymerase Chain Reaction) sequence analysis. The DNA was extracted by enzymatic lysis with proteinase K and Phenol-Chloroform. The Master Mix Colorless—Promega was used for reaction with the manufacturer's cycling conditions. The Integrated DNA Technologies (IDT) synthesized the primer following sequences below:

1. Forward: 5'-TACAGCCTGAGGACATATTA-3'
2. Reverse: 5'-GCACTTTAACCCTTCGATTAG-3'.

It is essential to state here that sex hormones determine sexual phenotype dimorphism in the fetal-programmed disease model in adulthood by changes in the long-term control of

neural, cardiac, and endocrine functions. Thus, the present study was limited and performed on male rats considering the findings above to eliminate interferences due to gender differences [5-7].

Real-time Quantitative PCR (mRNAs) - For the analysis of expression levels of NOS2, p53, HSP90, HIF-1α, NFκB, elF4, Ep300, TGFβ-1, mTOR, AT1a, AT1b, and AT2, in the isolated two-kidney pool, RT-qPCR was carried out with SYBR Green Master Mix, using primers specific for each gene, provided by Exxtend (Campinas, SP, Brazil) (Table 1). Reactions were set up in a total volume of 20 µL using 5 µl of cDNA (diluted 1:100), 10 µL SYBR Green Master Mix (Life Technologies, USA), and 2.5 µL of each specific primer (5 nM) and performed in the StepOnePlusTM Real-Time PCR System (Applied BiosystemsTM, USA). The cycling conditions were 95°C for 10 minutes, 45 cycles of 95°C for 15 seconds, and 60°C for 1 minute. Ct values were converted to relative expression values using the ΔΔCt method with offspring kidney data normalized to GAPDH as a reference gene.

Analysis of the Gene Expression - To analyze the differential expressions, the mRNA levels obtained for each gene (Table 1) were compared with the LP group concerning the appropriated NP group. Normalization of mRNA expression was made using the expression of the genes GAPDH. Relative gene expression was evaluated using the comparative quantification method. All relative quantifications were assessed using DataAssist software v 3.0, using the ΔΔCT method. PCR efficiencies calculated by linear regression from fluorescence increase in the exponential phase in the program LinRegPCR v 11.1 [5,7].

Immunohistochemistry – The fetus (n = 5 per group) was removed and immediately fixed in 4% paraformaldehyde (0.1 M phosphate, pH 7.4). The materials were dehydrated, diaphanized, and included in paraplast, and the blocks were cut into 5-µm-thickness sections. For immunohistochemistry, the paraffin sections were hydrated and washed in PBS (pH 7.2), and then the antigenic recovery was made with citrate buffer pH 6.0 for 25 minutes in the pressure cooker. Endogenous peroxidase was blocked with hydrogen peroxide and methanol for 10 minutes. For non-specific binding, the slides were incubated with a blocking solution (5% skimmed milk powder, in PBS) for 1 hour. The sections were incubated with the primary antibody (Table 2) and diluted in 1% BSA overnight in the refrigerator. After washing with PBS, the sections were exposed to the specific secondary antibody for 2 hours at room temperature. The slides were washed with PBS. The slices were revealed with DAB (3,3'-diaminobenzidine tetrahydrochloride, Sigma—Aldrich CO®, USA). After successive washing in

running water, the slides were counterstained with hematoxylin, dehydrated, and mounted with a coverslip using Entellan®. When the proteins studied had nuclear localization, the slides were not counterstained with hematoxylin in order not to cover the labeling. No immunoreactivity was seen in control experiments in which one of the primary antibodies was omitted Figure 1S.

Quantification - The immunohistochemistry quantification was performed by microscopic fields digitized (Olympus BX51) using CellSens Dimension. The studies were performed in a blinded and similar way for both groups of animals (NP and LP). In the metanephros section at its greatest longitudinal extent (% established for each group), all the CAP area was delimited, and the percentage of the marked area was automatically calculated.

References

Mesquita FF, Gontijo JA, Boer PA. Expression of renin-angiotensin system signalling compounds in maternal protein-restricted rats: effect on renal sodium excretion and blood pressure. Nephrol Dial Transplant 2010a; 25, 380-388. DOI: 10.1093/ndt/gfp505.

Mesquita FF, Gontijo JAR, Boer PA. Maternal undernutrition and the offspring kidney: from fetal to adult life. *Braz J Med Biol Res 2010b;* 2010b; 43, 1010–1018. DOI: 10.1590/s0100-879x2010007500113

Sene LB, Mesquita FF, de Moraes LN, Santos DC, Carvalho R, Gontijo JA, Boer PA. Involvement of renal corpuscle microRNA expression on epithelial-to-mesenchymal transition in maternal low protein diet in adult programmed rats. PLoS One. 2013; 19: e71310. https://doi.org/10.1371/journal.pone. 0071310 PMID: 23977013

Sene LB, Rizzi VHG, Gontijo JAR, Boer PA. (2018). Gestational low-protein intake enhances whole-kidney miR-192 and miR-200 family expression and epithelial-to-mesenchymal transition in rat adult male offspring. *J Exp Biol* 2018; 221:jeb171694. DOI: 10.1242/jeb.171694

Sene LB, Scarano WR, Zapparoli A, Gontijo JAR, Boer PA. Impact of gestational low-protein intake on embryonic kidney microRNA expression and in nephron progenitor cells of the male fetus. *PLoS One* 2021; 16:e0246289. DOI: 10.1371/journal.pone.0246289

Lamana GL, Ferrari ALL, Gontijo JAR, Boer PA. Gestational and breastfeeding low-protein intake on blood pressure, kidney structure, and renal function in male rat offspring in adulthood. *Front. Physiol 2021;* 12:658431 DOI: 10.3389/fphys.2021.658431

10. Results

The male 17GD LP offspring showed a significant reduction in the body mass compared to the age-matched NP group (Figure 1).

Gene expression analysis – The figure 2 shows the mRNA expression of the elected predicted gene expression analysis in metanephros of males in 17-DG. We observed a significant increase in eIF4, HSP90, p53, p300, NFκβ, and AT2 compared to age-matched NP (Figure 2). Regarding HIF-1α, the LP animals showed an increase in the expression of their mRNA concerning the NP. However, we did not observe statistical significance ($p=0.053$). The presentation of mRNAs encoding NOS2, AT1α, and AT1β, although AT2 receptors have been different between groups, was not altered in LP animals compared to control animals (Figure 2).

Immunohistochemistry – The HIF-1α is widely shown in cortical and medullar areas of the metanephros, preferentially located in the nuclear site associated with low cytosolic intensity (Figure 3). The CAP cells staining presents higher labeling in 17-DG offspring than in age-matched NP offspring (t=0,9669, df=85, p=0.0001). eIF4 immunoreactivity was dispersed in the cytosol of metanephros cell types (Figure 4). We observed a significant eIF4 reduction in LP progeny kidney tissue and CAP cells (t=5.838, df=101, p=0.0001). Also, the phosphorylated form of eIF4 staining was observed to reduce reactivity in the CAP cells (t=7,486, df=89, p=0.0001). Figure 4). In metanephros of NP animals, the HSP90 protein was located in a small quantity in the cytosol of different cell types. Otherwise, endothelial cells easily showed higher immunostaining to HSP90 (Figure 5). The immunoreactivity for HSP90 is higher in different metanephros nuclear cell types in LP offspring (t=6,770, df=115, p=0.0001). Quantification showed an increased percentage of marked CAP area in LP progeny compared to the NP offspring (Figure 5). In control animals (NP), the NFκβ staining is weak in all metanephric tissue (Figure 6). However, in 17DG LP, the NFκβ and nuclear immunoreactivity are significantly enhanced in all cells (t=2,822, df=117, p=0.0056). Also, the percentage of labeled CAP area was increased considerably in 17-DG LP (Figure 6). Although significantly increased in LP progeny metanephros, the NOS2 immunostaining occurred weakly throughout all metanephros extent (t=4,482, df=126, p=0.009, Figure 7). The VEGF

immunoreactivity was reduced in LP, and through the quantifications, we obtained a considerable reduction in the percentage of marked CAP area compared to NP progeny (Figure 8).

References

Mesquita FF, Gontijo JA, Boer PA. Expression of renin-angiotensin system signalling compounds in maternal protein-restricted rats: effect on renal sodium excretion and blood pressure. Nephrol Dial Transplant 2010a; 25, 380-388. DOI: 10.1093/ndt/gfp505.

Mesquita FF, Gontijo JAR, Boer PA. Maternal undernutrition and the offspring kidney: from fetal to adult life. *Braz J Med Biol Res 2010b;* 2010b; 43, 1010–1018. DOI: 10.1590/s0100-879x2010007500113

Sene LB, Mesquita FF, de Moraes LN, Santos DC, Carvalho R, Gontijo JA, Boer PA. Involvement of renal corpuscle microRNA expression on epithelial-to-mesenchymal transition in maternal low protein diet in adult programmed rats. PLoS One. 2013; 19: e71310. https://doi.org/10.1371/journal.pone. 0071310 PMID: 23977013

Sene LB, Rizzi VHG, Gontijo JAR, Boer PA. (2018). Gestational low-protein intake enhances whole-kidney miR-192 and miR-200 family expression and epithelial-to-mesenchymal transition in rat adult male offspring. *J Exp Biol* 2018; 221:jeb171694. DOI: 10.1242/jeb.171694

Sene LB, Scarano WR, Zapparoli A, Gontijo JAR, Boer PA. Impact of gestational low-protein intake on embryonic kidney microRNA expression and in nephron progenitor cells of the male fetus. *PLoS One* 2021; 16:e0246289. DOI: 10.1371/journal.pone.0246289

Lamana GL, Ferrari ALL, Gontijo JAR, Boer PA. Gestational and breastfeeding low-protein intake on blood pressure, kidney structure, and renal function in male rat offspring in adulthood. *Front. Physiol 2021;* 12:658431 DOI: 10.3389/fphys.2021.658431
