# Supplementary material for: Impact of maternal protein restriction on Hypoxia-Inducible Factor (HIF) expression in male fetal kidney development
Source: PLoS One. 2023 May 4;18(5):e0266293. doi: 10.1371/journal.pone.0266293 (PMC10159110; doi:10.1371/journal.pone.0266293)
Supplement: Attachment — Submitted filename: Final Response to Review Comments to the Author.docx [file pone.0266293.s005.docx]

LETTER TO EDITOR

                                                        	Campinas, Brazil, November 18, 2022

To

Emily Chenette, Editor-in-Chief

Plos One

Dear Editor:

I wish to submit an edited and revised version of the **manuscript** - titled “IMPACT OF maternal protein restriction on HYPOXIA-INDUCIBLE FACTOR (HIF) EXPRESSION IN MALE FETAL KIDNEY DEVELOPMENT” by Gomes et al. for publication in Plos One. I have read and have abided by the statement of ethical standards for documents submitted to the Plos One and the other information that all authors have approved the final article. Considering the severe consequences of maternal undernutrition on *offspring,* the current study data supported that nephron onset impairment in the 17DG fetus’s kidney, programmed by gestational low-protein intake, is, at least in part, related to alterations in the HIF-1α signaling pathway. Factors that facilitate the transposition of HIF-1α to the cell's nucleus, such as NOS, Ep300, and HSP90, may have an essential role in this regulatory process. This alteration leads to the inhibition of adaptive responses to the adverse environment. It is secondary to an increase in ungraded HIF-1α, possibly associated with a reduction in the transcription factor elF-4 and proteins of their respective signaling pathways. I am grateful for the reviewer's comments. After two evaluations by reviewers whose doubts, observations and comments were previously and now answered, I attach a response to the third reviewer, who made general considerations about the manuscript. The responses to the Reviewers are attached below.

Thank you for being so considerate. I look forward to hearing from you.

Sincerely yours,

José AR Gontijo, MD,

Campinas State University, Campinas, SP, Brazil.

E-mail: gontijo@fcm.unicamp.br

Response to Review Comments to the Author

**The general response from the authors:**

Thanks to the Reviewers for spending one more time and carefully reading and beware of the possible mistakes in this manuscript. We have greatly appreciated your comments and suggestions. As suggested, a native English speaker submitted the document for revision. The Introduction, Material, and Method and meanly Discussion sections of the manuscript were revised and completely rewritten to have the advice and comments of reviewer 1. Practically, whole manuscript sections were entirely rewritten, and reviewer suggestions were included in that new version.

**Reviewer #1**

Reviewer #1: The authors have addressed most of the points raised in my initial review; however, there are several issues that require further attention.

 1. Although the manuscript has been edited extensively, there are still instances where the English could be improved to aid clarity.

***Response from authors:*** As suggested, the manuscript was again submitted to a native English speaker for an edition.

2. I am not convinced by the argument put forward for only studying male fetuses. Sex is indeed a complication; however, it has been shown in numerous developmental programming studies that there are essential differences in the way that males and females respond to intrauterine challenges. Best practice now requires that both sexes are considered in studies of this nature.

***Response from authors:*** I appreciate the reviewer's comments on using male offspring only in the present study. Regarding the relevant question and observations of the Reviewer, It is essential to state here that sex hormones determine sexual phenotype dimorphism in the fetal-programmed disease model in adulthood by changes in the long-term control of neural, cardiac, and endocrine functions. Thus, the present study was limited and performed on male rats considering the findings above to eliminate interferences due to gender differences [Kwong et al., 2000; Gillette et al., 2017]. We now edited the manuscript text to include the justification above. We already have studies in progress, which involve the analysis of other organs (heart and central nervous system) in addition to the kidney, which has also been studying the impact of nutritional stress on the offspring of mice and rats. In the case of the study in females, we also included a group with induction of the estrous period. Additionally, as the reviewer emphasized, the neuro-humoral behavior of females is very peculiar and can modulate molecular and cellular responses differently.

3. Similarly, I am not convinced by the argument for only looking at gestational day 17. Nephrogenesis is a dynamic process that occurs pre- and postnatally in the rat. Selecting one gestational day provides a snapshot of development at that point, but it does not tell you if there were compensatory changes at a later stage. This should be recognized as a limitation in the discussion and the overall conclusions toned down.

***Response from authors:*** Thanks for reiterating the comments. The authors acknowledge that kidney development is a dynamic that occurs both pre- and postnatal in the rat. Taking into account the alterations in renal development already observed immediately after the birth of the offspring, we assume that the renal cellular and molecular alterations, particularly in the LP group, may be closely related to the reduction in the number of nephrons in this offspring. However, selecting an isolated gestational point may only partially reflect changes in renal development observed after a while. As suggested by the reviewer, we take care to include the following text in the conclusion of the manuscript...

Yes, we agree that complete maturation of the kidney ends in the postnatal period. However, we wanted to observe whether possible changes had already occurred before delivery, before the offspring's exposure to extra-uterine factors. Also, deliveries occur between 19 and 21 days frequently. The fetuses were randomly collected, including their position in the uterine horns, at the close time, at 17 gestational days for both experimental groups. An equal number of fetuses were taken from both uteri horn sides. The fetuses were removed and immediately euthanized by decapitation. The fetuses were weighed, and the tail and limbs were collected for sexing. Fetuses from half of the dams (5 for each group) metanephros was isolated and collected for Next Generation Sequencing (NGS), RT-qPCR, and the other half of fetuses were immersion fixed for immunohistochemistry analyses. Each litter was considered n = 1 to prevent litter effects from biasing or data analysis, and only one pup per litter was used for each experiment.

The renal developmental period can be influenced dramatically by alterations in the intrauterine environment that lead to impairment of nephrogenesis. Prior studies of our groups and other authors have demonstrated inappropriate renal development in gestational protein-restricted studies, such as reduction in nephron number, functional disorders, and hypertension in adulthood. In the present study, we consider that kidney development is an intricate process called branching morphogenesis, which involves several signaling molecules and transcription and growth factors. Any deregulation in this crucial process may lead to a change in cell proliferation apoptosis and impaired nephrogenesis. As correctly stated by the reviewer, the nephrogenesis in the rat starts on GD 13 and is not complete until postnatal day 10. The present study aims at 17 gestational days for both experimental groups; however, the kidney was not entirely developed. The histological analysis found a different pattern of development of nephrons, such as comma- and subsequent S-shaped with elongated bodies. Endothelial cells migrate into the distal end of the S-shaped body. But also, mature glomerulus is observed. Many primitive glomeruli differentiate, incorporating the vascular loops and allowing endothelial cells to contact visceral epithelial cells, forming the mature glomerulus's filtration barrier. So, at this gestational kidney developmental phase is possible already to observe kidney structures in the maturation stage and the effect of protein restriction in both offspring groups.

4. The authors have resolved some of the issues over presentation in figures 3-8; however, they have not clarified how the data presented in the graphs were collected. If I understand correctly, 5 NP and 5 LP litters were used for the immunostaining studies, and one male fetus from each litter was sampled. Looking at the scatter plots in figures 3-8, there are more than 5 data points for each group, which suggests that multiple sections have been taken from each kidney and individual data points have been analyzed. If that is the case, then the number of replicates is artificially inflated, affecting the statistical analysis as the number of technical replicates is much greater than the number of biological replicates. A better approach would be to quantify expression in n sections per kidney and then take the mean value of those n sections as representative of that individual kidney and fetus. That way, the number of data points entered into the analysis and represented in the scatter plot should equal 5 per group.

***Response from authors:*** Regarding the relevant question and comments of Reviewer 1, we have now rewritten the Methods section to read… **Area and cell analysis and Immunohistochemistry assessment** - The Hematoxylin-eosin stained paraffin sections (5NP and 5LP from different mothers) were used to measure kidney cortical and medullar areas from 17-GD offspring. The CAP areas and cell analysis were performed by microscopic fields digitized (Olympus BX51) using CellSens Dimension or ImageJ software evaluating images of the histological section in HE. In each half, the kidneys were sectioned longitudinally for this procedure and embedded in paraffin. Then, the microtome cuts were made after the block was trimmed and stained in HE. The studies were blinded and similar for both groups of animals (NP and LP) by digitized microscopic fields (Olympus BX51) using the CellSens Dimension. In the metanephros section at its greatest longitudinal extent (% established for each group), all the CAP area was delimited, and the percentage of the marked area was automatically calculated. The whole kidney area corresponds to the determination of measures of the entire cut, that is, the sum of the cortical and medullary area. Each histological section's five cortical and medullar fields were analyzed, and the average immunoreactivity reading was determined. The relative (%) of the cortical and medullary area related to the total renal area was also performed.

I am grateful for the reviewer's comments on the analysis of immunostaining*.* Both about the statistical analysis of results and the presentation of the figures*, we decided to keep it as initially.* However, statistically, it is not possible to say that an analysis procedure to measure the central tendency of a given sample and the variability of the points that compose it is more correct than another. Each analysis has different meanings, both approaches being proper in appropriate contexts. Thus,

a. In the present study, the author's idea was analyzed using the different points studying each histological section's five cortical and medullar fields, and the average immunoreactivity reading was determined. So, the results demonstrate the differences in the immunostaining of cells by area of CAPs of the other experimental groups and not the total immunostaining by kidneys.

b. Although taking the average of the means of immunostaining by CAP, similar values are obtained for the central point of the samples, there is an expressive variation of the variance at the different readings, expressed as standard deviation and standard error of the mean, which can interfere in the statistical analysis of the results.

**Reviewer #2**

Reviewer 2 felt completely satisfied with the authors' answers and arguments to the questions and doubts raised. No additional comments have been added.

**Reviewer #3**

**Reviewer #3:** This study is of potential interest to readers of PLOSone. Unfortunately, the manuscript is not well written. Writing style and English usage are not appropriate. Some of the study procedures are confusing. Data are not clearly presented. The bibliography regarding fetal programming is not complete (no paper from Barker). Relevant articles regarding human nephrogenesis and the putative role of hypoxia (Gerosa C et al., Int Urol Nephrol 2017, 49, 1621) are missing. Conclusions are difficult to read, confusing, not well written, and not completely supported by the data shown in this manuscript.

***Response from authors:*** Thanks to Reviewer 3 for spending time and carefully reading and beware of the possible mistakes in this manuscript. We have greatly appreciated your comments and suggestions. As suggested, a native English speaker submitted the document for revision. The Introduction, Material, and Method and meanly Discussion sections of the manuscript were revised and completely rewritten to have the advice and comments of reviewer 1. Practically, whole manuscript sections were entirely rewritten, and reviewer suggestions were included in that new version. We also included in the text the reference [24] as suggested by reviewer Gerosa C, Fanni D, Faa A., et al. Low vascularization of the nephrogenic zone of the fetal kidney suggests a major role for hypoxia in human nephrogenesis. Int Urol Nephrol 2017; **49**, 1621–1625 (2017). https://doi.org/10.1007/s11255-017-1630-y.

Of course, we also include quotes from Barker et al., pioneers in the study of fetal programming.
