# Supplementary material for: Impact of maternal protein restriction on Hypoxia-Inducible Factor (HIF) expression in male fetal kidney development
Source: PLoS One. 2023 May 4;18(5):e0266293. doi: 10.1371/journal.pone.0266293 (PMC10159110; doi:10.1371/journal.pone.0266293)
Supplement: Attachment — Submitted filename: Final Response to Review Comments to the Author.docx [file pone.0266293.s006.docx]

LETTER TO EDITOR

                                                        	Campinas, Brazil, March 10, 2023

To

Emily Chenette, Editor-in-Chief

Plos One

Dear Editor:

We submit the edited and revised version of the **manuscript** - titled “PONE-D-22-07908R2

IMPACT OF MATERNAL PROTEIN RESTRICTION ON HYPOXIA-INDUCIBLE FACTOR (HIF) EXPRESSION IN MALE FETAL KIDNEY DEVELOPMENT” by Gomes et al. for publication in Plos One. I have read and have abided by the statement of ethical standards for documents submitted to the Plos One and the other information that all authors have approved the final article. Considering the severe consequences of maternal undernutrition on *offspring,* the current study data supported that nephron onset impairment in the 17DG fetus’s kidney, programmed by gestational low-protein intake, is, at least in part, related to alterations in the HIF-1α signaling pathway. Factors that facilitate the transposition of HIF-1α to the cell's nucleus, such as NOS, Ep300, and HSP90, may have an essential role in this regulatory process. This alteration leads to the inhibition of adaptive responses to the adverse environment. It is secondary to an increase in ungraded HIF-1α, possibly associated with a reduction in the transcription factor elF-4 and proteins of their respective signaling pathways. I am grateful for the reviewer's comments. After evaluations by reviewers whose doubts, observations and comments were previously and now answered, I attach a response to the third reviewer, who made general considerations about the manuscript. The responses to the Reviewers are attached below.

Thank you for being so considerate. I look forward to hearing from you.

Sincerely yours,

José AR Gontijo, MD,

Campinas State University, Campinas, SP, Brazil.

E-mail: gontijo@fcm.unicamp.br

Response to Review Comments to the Author

PONE-D-22-07908R2

IMPACT OF MATERNAL PROTEIN RESTRICTION ON HYPOXIA-INDUCIBLE FACTOR (HIF) EXPRESSION IN MALE FETAL KIDNEY DEVELOPMENT

**The general response from the authors:**

Thanks to the Reviewers for spending one more time and carefully reading and beware of the possible mistakes in this manuscript. We have greatly appreciated your comments and suggestions. As suggested, a native English speaker submitted the document for revision. The Abstract, Introduction, Material, Method, and Discussion sections were revised and completely rewritten to have the advice and comments of reviewers in that new version.

**Reviewer #1**

Reviewer #1: No comments

**Reviewer #2**

Reviewer 2 felt completely satisfied with the authors' answers and arguments to the questions and doubts raised. No additional comments have been added.

**Reviewer #3**

Reviewer 3: the authors are encouraged to extensively re-write their manuscript. This last version of the paper is characterized by too many errors and mistakes. The bad English does not allow the rider to understand the meaning of too many sentences. Moreover, the fusion of the results and conclusions, in the absence of a discussion, in the abstract is not correct. The authors are encouraged to summarize the most important results and, subsequently, to discuss them on the basis of previous data reported in the literature. Some terms like “cuts”, not used in the laboratory practice, should be deleted. “Sections” might be much more appropriate. In the section “Materials and methods” some sentences should be accurately revised by a pathologist or by a technician with expertise in immunohistochemistry. To use eosin in sections immunostained for antigens expressed in the cytoplasm is not appropriate.

**Response from authors:** Thanks to Reviewer 3 for spending time and carefully reading and beware of the possible mistakes in this manuscript. We have greatly appreciated your comments and suggestions. As suggested, a native English speaker submitted the document for revision. The Abstract, Introduction, Discussion and particularly Material and Method sections of the manuscript were revised and completely rewritten to have the advice and comments of reviewer

1. Practically, whole manuscript sections were entirely rewritten, and reviewer suggestions were included in that new version. We also included in the text the reference [24] as suggested by reviewer Gerosa C, Fanni D, Faa A., et al. Low vascularization of the nephrogenic zone of the fetal kidney suggests a major role for hypoxia in human nephrogenesis. Int Urol Nephrol 2017; **49**, 1621–1625 (2017). https://doi.org/10.1007/s11255-017-1630-y.

Of course, we also include quotes from Barker et al., pioneers in the study of fetal programming.
